# Effect of Surface Wettability on Nanoparticle Deposition during Pool Boiling on Laser-Textured Copper Surfaces

**DOI:** 10.3390/nano14030311

**Published:** 2024-02-04

**Authors:** Jure Berce, Armin Hadžić, Matic Može, Klara Arhar, Henrik Gjerkeš, Matevž Zupančič, Iztok Golobič

**Affiliations:** 1 Faculty of Mechanical Engineering, University of Ljubljana, Aškerčeva 6, 1000 Ljubljana, Slovenia; jure.berce@fs.uni-lj.si (J.B.); armin.hadzic@fs.uni-lj.si (A.H.); matic.moze@fs.uni-lj.si (M.M.); klara.arhar@fs.uni-lj.si (K.A.); matevz.zupancic@fs.uni-lj.si (M.Z.); 2 School of Engineering and Management, University of Nova Gorica, Vipavska 13, 5000 Nova Gorica, Slovenia; henrik.gjerkes@ung.si

**Keywords:** nanoparticles, nanoparticle deposition, nanofluids, laser texturing, hydrophobization, boiling heat transfer

## Abstract

Prior studies have evidenced the potential for enhancing boiling heat transfer through modifications of surface or fluid properties. The deployment of nanofluids in pool boiling systems is challenging due to the deposition of nanoparticles on structured surfaces, which may result in performance deterioration. This study addresses the use of TiO_2_–water nanofluids (mass concentrations of 0.001 wt.% and 0.1 wt.%) in pool boiling heat transfer and concurrent mitigation of nanoparticle deposition on superhydrophobic laser-textured copper surfaces. Samples, modified through nanosecond laser texturing, were subjected to boiling in an as-prepared superhydrophilic (SHPI) state and in a superhydrophobic state (SHPO) following hydrophobization with a self-assembled monolayer of fluorinated silane. The boiling performance assessment involved five consecutive boiling curve runs under saturated conditions at atmospheric pressure. Results on superhydrophilic surfaces reveal that the use of nanofluids always led to a deterioration of the heat transfer coefficient (up to 90%) compared to pure water due to high nanoparticle deposition. The latter was largely mitigated on superhydrophobic surfaces, yet their performance was still inferior to that of the same surface in water. On the other hand, CHF values of 1209 kW m^−2^ and 1462 kW m^−2^ were recorded at 0.1 wt.% concentration on both superhydrophobic and superhydrophilic surfaces, respectively, representing a slight enhancement of 16% and 27% compared to the results obtained on their counterparts investigated in water.

## 1. Introduction

Pool boiling, recognized for its exceptional efficacy of heat dissipation, has found application in various industrial systems, including air conditioning [1,2], heat exchangers [3,4,5], domestic refrigerators [6,7], spacecraft thermal management [8,9], and high-power electronics cooling [10,11,12,13]. In recent decades, various cooling methodologies have been advanced for the purpose of heat dissipation, encompassing techniques involving porous media, microchannel heat sinks, spray cooling, as well as natural and forced convection, among others. Among these approaches, pool boiling has emerged as a notably efficient method, demonstrating superior heat dissipation efficacy compared to prevalent contemporary cooling techniques. Notably, a select few, namely, spray cooling, microchannel heat sinks, and heat removal techniques involving porous media, exhibit comparable or slightly lower efficiency levels within the spectrum of contemporary cooling technologies [13,14,15]. However, the present demands of increased heat removal capacities, coupled with the imperative for enhanced efficiency in heat transfer systems, give rise to a notable challenge. In response to this challenge, recent research efforts have concentrated on augmenting key parameters of boiling heat transfer, specifically the heat transfer coefficient (HTC) and critical heat flux (CHF). The latter represents the upper limit of nucleate boiling, manifesting when the surface is covered with an excessive population of bubbles at high heat flux, leading to extensive coalescence and the formation of an insulating vapor blanket. This blanket significantly reduces heat transfer intensity. The heat transfer coefficient is defined as the ratio between dissipated heat flux and the corresponding wall superheat, denoting the temperature differential between the boiling surface and the bulk fluid. HTC is calculated by the following equation:

(1) $$h=\frac{\dot{q}}{T_{w}-T_{sat}}$$

In Equation (1), $\dot{q}$ presents the heat flux, while *T_w_* presents the calculated surface temperature and *T_sat_* presents the saturation temperature of the bulk fluid.

To enhance boiling performance, one method involves manipulating the thermophysical properties of the boiling fluid, often achieved by adding nanoparticles to the base fluid, thereby forming nanofluids [16,17]. While certain studies have demonstrated improved boiling performance with nanofluids, challenges arise in controlling the thickness of deposited nanoparticles on surfaces, causing added thermal resistance and deterioration of heat transfer, resembling particulate fouling [18,19]. Addressing this, various parameters such as nanoparticle concentration [20,21], type [22,23], size [24], and deposition time during boiling [25,26] have been investigated to optimize heat transfer performance. Higher concentrations result in a reduction in HTC, attributed to elevated thermal resistance due to the growth of the deposited layer on the surface [27,28]. Furthermore, studies focused on the investigation of the effect of nanofluid boiling time demonstrated that an increase in nanofluid boiling time results in the formation of a deposited nanoparticle layer, leading to a reduction in contact angle and an increase in CHF [16,29]. However, it was also reported that the deposition of nanoparticles on the heated surface decreased the boiling HTC due to the rise in thermal resistance [15,29].

In addition to concentration and deposition time, the material and size of nanoparticles, as well as nanofluid preparation, influence boiling efficiency. Dadhich et al. [30] and Fang et al. [28] highlighted the considerable necessity for a comprehensive database encompassing thermal properties across various materials and nanoparticle size ranges. Moreover, we previously showed [29] that nanofluids with small (4–8 nm) and large (490 nm) nanoparticles lead to the deterioration of HTC on copper surfaces with laser-induced structure in comparison to the results obtained with pure water on laser-engineered surfaces. The deterioration of HTC was more pronounced with the utilization of larger nanoparticles, which was mainly attributed to the filling of laser-induced microcavities with particles that are approximately similar in size. One of the most promising ways to utilize nanofluid to have a positive effect on heat transfer efficiency in boiling systems is to reduce the deposition rates of nanoparticles on boiling surfaces.

Furthermore, a more promising method for enhancing boiling heat transfer involves tailoring surface morphology and topology. Various techniques, including electrochemical processes [31,32], chemical vapor deposition [33], laser texturing [34,35], and wet/dry etching [36], have been employed to fabricate surfaces with porous structures [37], micropin fins [38], nanowire and nanoflower structures [39], and microcavities [40] on boiling interfaces. Surfaces with tailored interfaces have demonstrated increased CHF due to augmented liquid supply. Moreover, altering surface wettability has been explored to improve liquid supply, thereby enhancing CHF and HTC. Hydrophilic surfaces have been associated with higher CHF values, while surfaces with opposite wetting properties reduce the energy barrier for nucleate boiling onset, leading to improved HTCs, especially at low and medium heat fluxes. However, such surfaces are incapable of limiting bubble merging, which leads to a lower CHF value compared to surfaces with better wetting properties. Nevertheless, recent research findings presented by Allred et al. [41] and Može et al. [40] demonstrate that significant enhancement of HTC can be achieved on adequately degassed superhydrophobic surfaces, without causing a drastic reduction in CHF. Furthermore, surfaces denoted as “(super)biphilic” that exhibit heterogeneous wettability with juxtaposed hydrophilic and hydrophobic regions have the aim of manipulating nucleation phenomena while considering the interplay of liquid and vapor flows, which can lead to simultaneous enhancement of HTC and CHF [42,43,44,45].

The surfaces with tailored wettability and their effect on boiling performance are mostly tied to tests with pure water. Although there are several studies that have tested nanofluid boiling performance on structured surfaces with tailored wetting properties, structured surfaces that exhibited a superhydrophilic wetting state demonstrated the greatest critical heat flux, but also the deterioration of HTC, due to filling the artificial cavities with nanoparticles, resulting in reduced nucleation site density [29,46]. Furthermore, Freitas et al. [47] investigated the pool boiling performance and bubble dynamics of nanofluids on biphilic surfaces. The low-wettable areas enable the control of bubble merging, while the highly wettable surroundings promote the liquid supply, contributing to the cooling of the surface. Furthermore, they showed that low-concentrated nanofluids have a minor influence on bubble dynamics and heat transfer efficiency during boiling. Ultimately, a single study, published by Ji et al. [48], was identified as having previously integrated surfaces possessing hydrophobic and superhydrophobic properties with the utilization of nanofluids to augment boiling heat transfer. For the superhydrophobic surface with higher roughness (contact angle 153.0°), boiling heat transfer was enhanced at a heat flux less than 93 kW m^−2^, and then the heat transfer degraded at a higher heat flux.

There is limited research examining the boiling performance of nanofluids on laser-functionalized surfaces with tailored wettability, despite the seemingly great potential for enhancement of boiling performance and the possibility of mitigating the deposition of nanoparticles during nanofluid boiling by tailoring the wettability of the structured surfaces. In an effort to address this gap, we conducted an examination of the pool boiling performance of TiO_2_–water nanofluids at two mass concentrations (0.001 and 0.1 wt.%) of nanoparticle sizes of 4–8 nm, on superhydrophilic and superhydrophobic laser-textured copper surfaces. Five consecutive pool boiling curve measurements were performed on each surface at atmospheric pressure. Laser-functionalized surfaces were developed utilizing nanosecond laser texturing, followed by the application of hydrophobic monolayer coating using a 3 mM solution of 2-propanol and fluorinated alkyl phosphonic acid. To clarify the effect of nanoparticle deposition on boiling performance, surface characteristics after boiling tests with nanofluids were analyzed using contact angle measurements and scanning electron microscopy.

## 2. Materials and Methods

### 2.1. Surface Preparation and Analysis

High-purity copper samples (>99.9%) were used to perform the pool boiling experiments. The top part of the samples served as a boiling interface, which was first sanded with sandpapers P1200 and P2000, and then cleaned with 2-propanol prior to testing or subsequent treatment. The achieved roughness (*Ra*) of the sanded samples was approximately 0.2 μm. The surface roughness of the tested samples was measured using a Mitutoyo SJ-310 device (Mitutoyo Corporation, Kanagawa, Japan). Among the tested samples, one was tested without any further treatment and served as a reference surface, while other samples underwent further functionalization of the boiling interface.

The first step in functionalizing surfaces was laser texturing performed with a nanosecond fiber laser (JPT Opto-electronics Co., Ltd., Shenzhen, China, M7 30 W MOPA; *λ* = 1064 nm). The laser beam was directed across the surface with an F-Theta lens with a working area of 70 × 70 mm^2^ and a focal distance of 100 mm. The focused beam spot diameter was approx. 25 μm, the laser beam quality M^2^ < 1.3 (manufacturer data), and the maximal power of the laser source was 30 W. Surface treatment patterns were designed in ezCAD 2 software, which was also used to control the laser system during the laser treatment. The microstructure on surfaces was fabricated utilizing a pattern of equidistant parallel lines (Δx = 60 μm) with the following laser processing parameters: pulse duration of 45 ns, pulse frequency of 110 kHz, and maximum laser power of 30 W.

Three samples after laser texturing underwent further functionalization that included hydrophobization utilizing a solution of 3,3,4,4,5,5,6,6,7,7,8,8,9,9,10,10,11,11,12,12,12-henicosafluorododecylphosphonic acid (abbreviated to FDPA, CAS Nr. 252237-39-1, abcr GmbH product code AB254085). FDPA was dissolved in 2-propanol (Honeywell, ACS reagent, Charlotte, NC, USA, ≥99.5%) at a concentration of 3 mM. The hydrophobization process of the surfaces was performed by pipetting three drops of FDPA solution onto the sample. Furthermore, samples were left to dry at room temperature for 5 min, then in the oven at 80 °C for another 15 min.

The collection of surfaces and boiling fluids prepared and tested within the study is summarized in Table 1.

**Figure 1:**
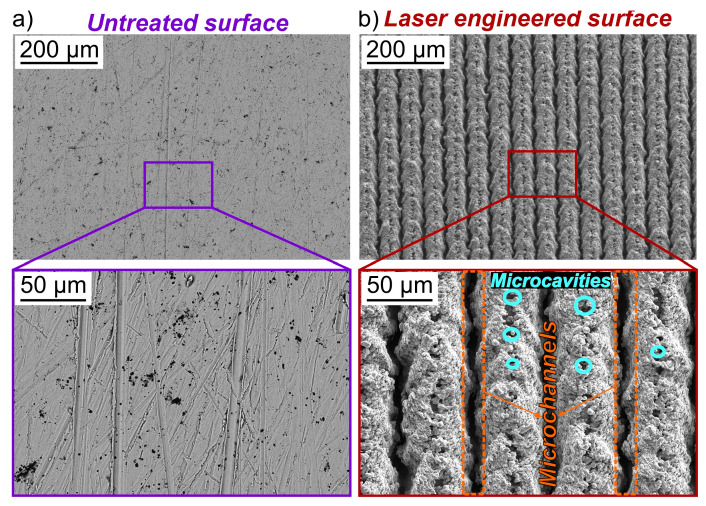
SEM images of the reference samples: (**a**) untreated surface and (**b**) laser-engineered surface.

The examination of functionalized surface properties involved utilizing scanning electron microscopy (SEM) and contact angle measurements. To analyze the micro- and nanostructure of the surfaces, a scanning electron microscope (ThermoFisher Scientific Quattro S, Waltham, MA, USA) was employed, employing both backscattered electron (BSE) and secondary electron (SE) imaging at an accelerating voltage of 10 kV. Figure 1 shows SEM images of the untreated and laser-engineered surfaces before they underwent boiling tests. Contact angle measurements were conducted using a goniometer (Ossila, Sheffield, UK), and the assessments were carried out with twice-distilled water at room temperature. These measurements were performed on the prepared and tested samples both immediately before and after the boiling test within the scope of this study. The static contact angle results for the tested surfaces are presented in Table 1.

### 2.2. Nanofluid Preparation

The titanium dioxide-water nanofluids employed in this investigation were prepared using twice-distilled water, previously degassed utilizing vigorous boiling, and TiO_2_ nanoparticles. The stability of nanofluid solutions was maintained by utilizing ultrasonic vibration. The selection of TiO_2_ nanoparticles for this study was motivated by their commendable chemical and physical stability, coupled with their hydrophilic properties, previously observed to augment the critical heat flux (CHF) value in boiling experiments, which could be helpful to the augmentation of CHF on superhydrophobic surfaces, due to their known characteristic of decreasing the CHF.

The nanofluid solutions consisted of small nanoparticles, ranging in size from 4 to 8 nm (Carl Roth, Karlsruhe, Germany; ROTInanoMETIC ≥ 99.9%), and were prepared at two distinct mass concentrations: 0.1 and 0.001 wt.%. Before the preparation of each solution, 100 mL of twice-distilled water underwent degassing for 45 min through vigorous boiling to mitigate the presence of non-condensable gases. In the first step of nanofluid preparation for both concentrations, twice the amount of nanoparticles was dispersed in 100 mL of degassed twice-distilled water. Subsequently, the nanofluids underwent one hour of sonication immediately after preparation in an ultrasonic bath (ASonic, Ultrasonic Clean-er-Pro 30, 40 kHz, 120 W) to ensure homogeneity and mitigate the potential for irregularities in the distribution of nanoparticles. The other 100 mL of twice-distilled water was used for the degassing of the superhydrophobic surface and removing entrapped gasses from surface cavities. This protocol of degassing both the surface and the solution removed the need for using the nanofluid solution for degassing within the boiling chamber and the possible reduction of nanoparticle concentration due to their deposition on the immersion heater. The final nanofluid solutions of 200 mL with concentrations of 0.001 wt.% and 0.1 wt.% were produced by mixing 100 mL of double-concentrated, previously prepared nanofluid solutions into 100 mL of twice-distilled water within the boiling chamber.

### 2.3. Experimental Setup

**Figure 2:**
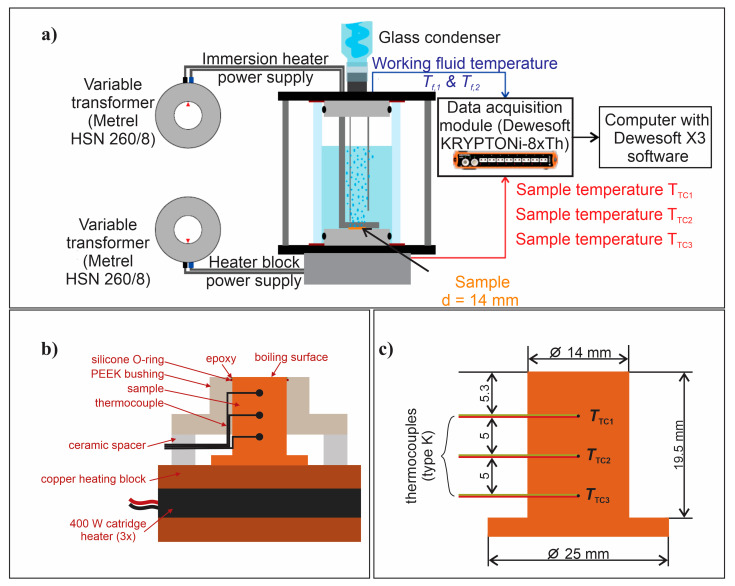
(**a**) Experimental setup; (**b**) cross section of the sample and the heating assembly; and (**c**) dimensions of the sample, including the position of thermocouples.

The assessment of boiling performance on laser-textured surfaces with tailored wettability was conducted through the utilization of a previously established experimental setup, depicted in Figure 2. Boiling tests were performed utilizing a glass boiling chamber with an inner diameter of 60 mm. The working fluid temperature was monitored using two submerged K-type thermocouples at varying heights within the chamber. A condenser, cooled by a secondary water loop, was positioned atop the assembly to maintain a consistent water level during experiments. Temperature signals were recorded as raw voltages at a rate of 10 Hz, employing a DAQ device (KRYPTIONi-8xTH; Dewesoft, Trbovlje, Slovenia) and Dewesoft X3 2022.1 software. Further details about the experimental setup can be found in our previous work [29,43].

### 2.4. Data Reduction and Measurement Uncertainty

**Figure 3:**
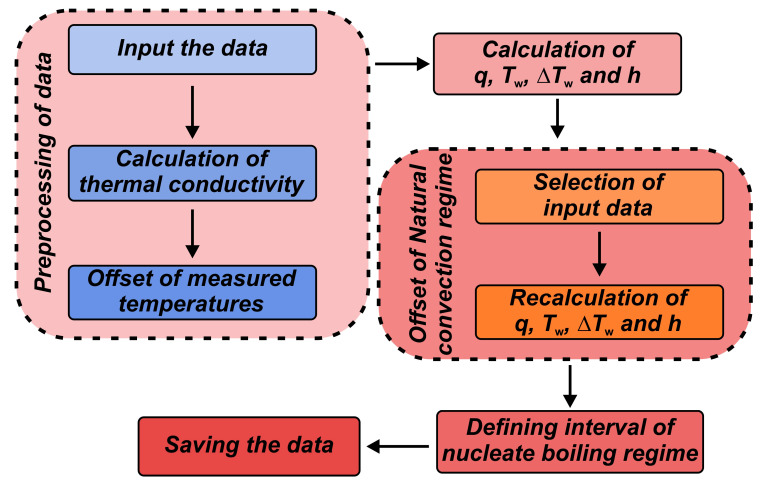
Flowchart of processing the data obtained during pool measurements.

Altogether, five temperatures were measured during the experiment—three inside the sample and two in the water. From these, the heat flux, surface superheat, and heat transfer coefficient were calculated and recorded in real time with the DewesoftX3 2022.1 software, while the post-processing was conducted with Mathworks MATLAB R2021a. The flowchart of data post-processing conducted with MATLAB is presented in Figure 3. More details are available in our previous work [43,49].

Briefly, the temperature-dependent thermal conductivity was determined at the average sample temperature, calculated as the arithmetic mean of three thermocouple measurements. The heat flux was computed using Fourier’s law of one-dimensional heat conduction, utilizing the temperature gradient derived from the thermocouple readings. Surface superheat was derived as the difference between the sample surface and water temperatures. The latter was determined as the arithmetic mean of both submerged thermocouple measurements, while the surface temperature was estimated using the measurement closest to the surface (upper sample thermocouple) and the heat flux. The heat transfer coefficient was then obtained by dividing the heat flux by the surface superheat.

The measurement uncertainty of heat transfer parameters is presented in Table 2.

### 2.5. Measurement Procedure

Boiling experiments for every combination of working fluid and surface featuring customized wettability comprised five runs. Each run involved recording the boiling curves up to the CHF, spanning a duration of 45 min. Throughout the boiling curve recording, the heat flux was slowly increased, following the methodology outlined by Može et al. [50]. Upon reaching CHF, the experimental run was completed, and the surface was allowed to cool down below 90 °C, which lasted approximately 15 min. Overall, the whole boiling test of a given sample was completed in a 5 h period.

## 3. Results

### 3.1. Pool Boiling Heat Transfer in Water

**Figure 4:**
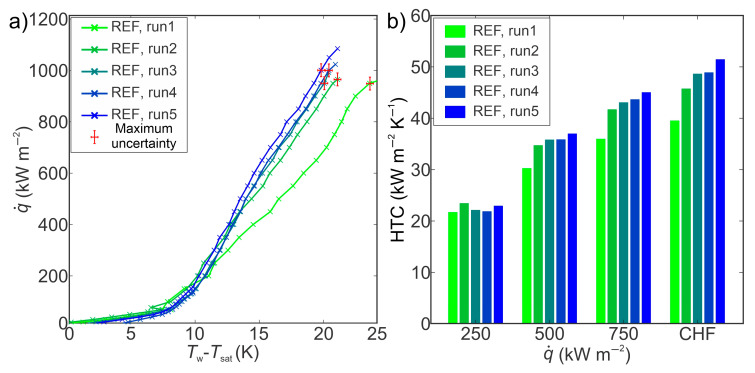
(**a**) Boiling curves and (**b**) heat transfer coefficients on the reference surface.

The evaluation of the boiling performance of the reference surface (REF) using twice-distilled water as a working fluid is shown in Figure 4. The untreated reference surface represents the initial state prior to any further functionalization. The highest CHF on untreated surfaces was 1084 kW m^−2^ and the highest HTC of 51 kW m^−2^ K^−1^ at CHF was recorded during the fifth experimental run. During five repetitions of boiling tests on untreated surfaces, the shift of boiling curves towards lower superheat was observed. A 4.3 K reduction of surface superheat at high heat flux level (800 kW m^−2^) near CHF was recorded, which can be attributed to the change of wetting state of the reference surface, i.e., from hydrophilic (contact angle of 61°) to hydrophobic (contact angle of 93°) after exposure to boiling.

**Figure 5:**
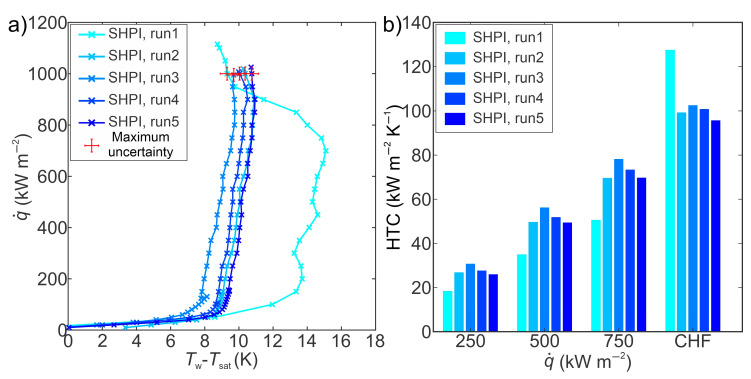
(**a**) Boiling curves and (**b**) heat transfer coefficients on the superhydrophilic laser-textured surface.

Figure 5 shows the boiling performance (Figure 5a—boiling curves and Figure 5b—heat transfer coefficients at different heat flux levels) obtained on a superhydrophilic laser-engineered surface. Laser texturing of untreated copper surfaces forms microchannels and microcavities, which can serve to enhance liquid supply and increase nucleation site density, respectively [43,51]. The highest CHF of 1152 kW m^−2^ was recorded during the first experimental run. At CHF, the largest HTC value of 128 kW m^−2^ K^−1^ was recorded, and an enhancement over the reference surface of 150% was calculated. The remarkable enhancement of HTC can be attributed to the occurrence of secondary boiling effects near the CHF, leading to a significant reduction of surface superheat. The incipience of secondary boiling effects can be the result of the increase in the number of nucleation sites at high heat flux levels, where bubbles begin to nucleate not just within microcavities, but also on the peaks of the laser structure [43,52]. Moreover, the shift of the boiling curve towards lower superheats was also observed, with a 3.3 K reduction of superheat recorded at heat flux near the CHF (800 kW m^−2^). This is the result of surface chemistry transformation occurring at high temperatures after reaching the first CHF during film boiling, as shown by Može et al. [53].

**Figure 6:**
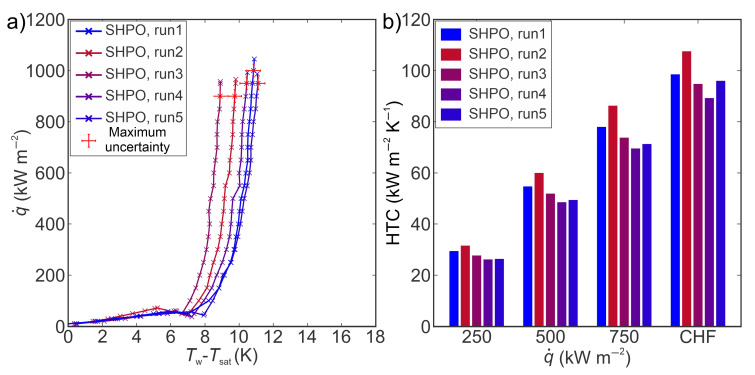
(**a**) Boiling curves and (**b**) heat transfer coefficients on the superhydrophobic laser-textured surface.

Figure 6 shows the boiling performance (Figure 6a—boiling curves and Figure 6b—HTCs at different heat flux levels) obtained on a superhydrophobic laser-engineered surface. Laser-induced cavities on the surface act as favored locations for nucleation during the boiling process, especially after the hydrophobization process. The static contact angle of 164° ± 0.7° was recorded on superhydrophobic surfaces before boiling tests, while after exposure to boiling, a value of 140° ± 0.8° was measured. The highest HTC of 108 kW m^−2^ K^−1^ at CHF was obtained during the third experimental run, with an enhancement of 112% over the reference surface. The average HTC of 97 kW m^−2^ K^−1^, obtained on superhydrophobic surfaces during five boiling tests, was similar to the average HTC (99 kW m^−2^ K^−1^) recorded on a superhydrophilic (SHPI) surface. The shift of boiling curves towards lower superheat was also observed on the superhydrophobic (SHPO) surface, with a 0.2 K reduction of surface superheat at a heat flux of 500 kW m^−2^ from the first to the fifth experimental run. On the other hand, at the same heat flux values, a 3.9 K reduction of superheat was recorded on a superhydrophilic surface, indicating that the SHPO sample is significantly more stable than the SHPI surface. The CHF values during the five boiling tests were similar to the CHF values obtained on the reference surface.

### 3.2. Boiling Heat Transfer on Superhydrophilic Surfaces with TiO_2_–Water Nanofluid

**Figure 7:**
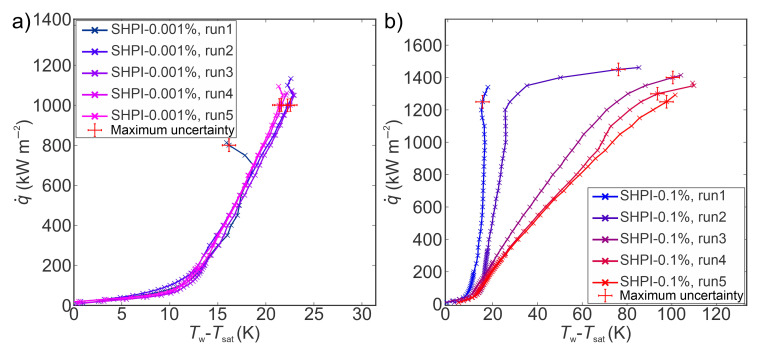
Boiling curves of TiO_2_–water nanofluid on a superhydrophilic laser-textured surface at (**a**) 0.001 wt.% and (**b**) 0.1 wt.% concentrations of TiO_2_ nanoparticles.

Boiling curves recorded on a superhydrophilic laser-engineered surface using TiO_2_ water nanofluid at two distinct concentrations are shown in Figure 7. Boiling curves obtained with lower concentrations were stable, and an increase in CHF of 35% from the first to the fifth experimental run was observed. On the other hand, boiling curves obtained with higher concentrations were unstable, and they shifted toward higher superheats with a low increase of CHF from the first to the second experimental run, followed by a decrease from the second to the fifth experimental run. The shift towards higher superheats obtained for the 0.1 wt.% nanofluid can be attributed to decreasing nucleation site density due to the deposition of nanoparticles in microcavities with each consecutive run. At high nanoparticle concentrations, the deposited layer on the surface becomes thicker, which leads to greater thermal resistance and decreases the HTC. However, the highest CHF of 1462 kW m^−2^ was recorded at higher concentrations, representing an improvement of 35% compared to the highest CHF obtained on the reference sample. The CHF values obtained at 0.1 wt.% were higher compared to the CHF values achieved at 0.001 wt.%, which can be attributed to better wetting properties (i.e., the static contact angle recorded on the surface after boiling with 0.1 wt.% nanofluid was around 3°, while on the surface after boiling, 0.001% wt.% nanofluid was 110°). The observed effect of concentration on CHF is in agreement with the findings presented in the literature [29,54].

**Figure 8:**
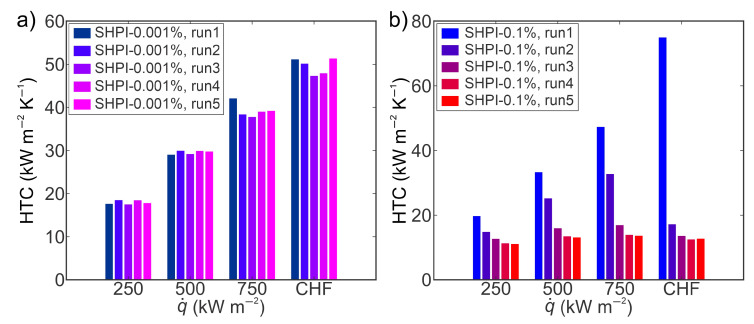
Heat transfer coefficients of TiO_2_–water nanofluid on a superhydrophilic laser-textured surface at (**a**) 0.001 wt.% and (**b**) 0.1 wt.% concentrations of TiO_2_ nanoparticles.

Heat transfer coefficients obtained at various heat flux levels on superhydrophilic laser-engineered surfaces employing TiO_2_–water nanofluid at 0.001 and 0.1 wt.% concentrations are presented in Figure 8. The findings indicate that the heat transfer coefficients during five consecutive experimental runs at 0.001 wt.% remain remarkably consistent, exhibiting no discernible alterations. In contrast, the boiling performance at 0.1 wt.% shows a pronounced deterioration from the first to the fifth run. This decrease can be predominantly attributed to a reduction in nucleation site density on the structured surface, stemming from the deposition of nanoparticles onto the surface, or more precisely, filling the laser-induced microcavities, and thus influencing boiling performance. Furthermore, the disparities in boiling performance over an extended boiling duration at different concentrations can be attributed to the quantity of nanoparticles present in the nanofluid capable of being deposited onto the surface during boiling. Consequently, the initial experimental run at 0.1 wt.% reveals an increased deposition of nanoparticles on the surface, effectively filling more laser-induced cavities and forming a thicker nanoparticle layer compared to the surface after the first experimental run at 0.001 wt.%. Following five hours of boiling at both concentrations, a substantial contrast in the deposited nanoparticle layers on the tested surfaces became apparent.

**Figure 9:**
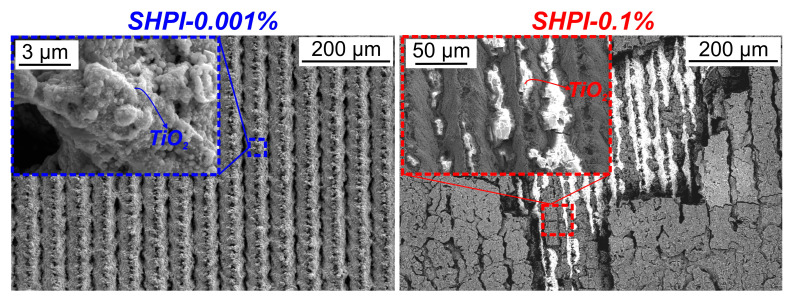
SEM images of superhydrophilic samples after boiling at concentrations of 0.001 wt.% (**left**) and 0.1 wt.% (**right**).

Corroborating this, the conducted SEM analysis (Figure 9) demonstrates that a very thin deposited layer of TiO_2_ nanoparticles persists on the laser-engineered structure after five hours of boiling at 0.001 wt.% and is only evident at higher magnifications. Conversely, during boiling at higher concentrations (0.1 wt.%), the deposited nanoparticles on the surface manifest more evidently, filling a substantial portion of the laser-engineered surface channels and forming a thicker nanoparticle layer. Thickening of the deposit on the surface results in additional thermal resistance, which can also result in the deterioration of the heat transfer coefficient. Similar observations of the effect of concentration on the deposited layer and further on boiling performance can be found in the literature [27,55,56].

### 3.3. Boiling Heat Transfer on Superhydrophobic Surfaces with TiO_2_–Water Nanofluid

**Figure 10:**
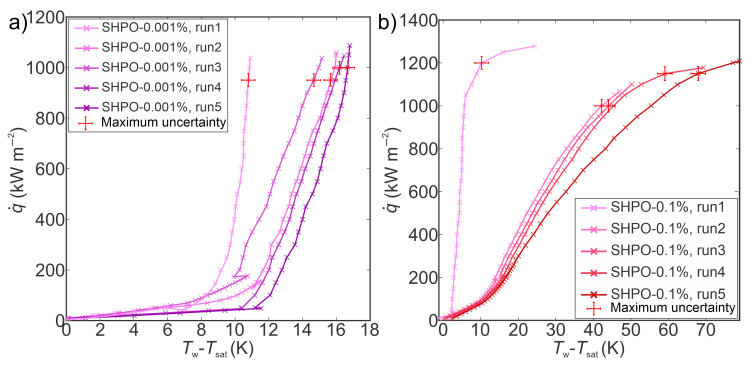
Boiling curves of TiO_2_–water nanofluid on a superhydrophobic laser-textured surface at (**a**) 0.001 wt.% and (**b**) 0.1 wt.% concentrations of TiO_2_ nanoparticles.

The boiling performance of TiO_2_ nanofluid (at concentrations of 0.001 wt.% and 0.1 wt.%) on superhydrophobic laser-engineered surfaces was assessed employing the same approach as that applied to their superhydrophilic counterparts. Five boiling curves were recorded up to CHF incipience for both concentrations, resulting in five-hour boiling tests for each concentration on superhydrophobic samples. The boiling curves corresponding to a concentration of 0.001 wt.% are shown in Figure 10a, while those for a concentration of 0.1 wt.% are illustrated in Figure 10b. It was observed that the boiling curves acquired on superhydrophobic surfaces with nanofluids were unstable, exhibiting a shift toward higher superheats after the initial experimental run. The shift of boiling curves with each consecutive run is the result of a decrease in nucleation site density that can be attributed to the deposition of nanoparticles that were filling the microcavities. The CHF values demonstrated an upward trend from the first to the fifth run on superhydrophobic surfaces at both concentrations, achieving the most substantial increase of 19% at 0.1 wt%. This increase in CHF is ascribed to a thicker and more porous layer that formed on superhydrophobic surfaces due to the deposition of hydrophilic TiO_2_ nanoparticles with each following run. The deposited hydrophilic nanoparticles covering the superhydrophobic surface result in the transition of wetting from superhydrophobicity towards hydrophilicity. The latter was confirmed by contact angle measurements after boiling, where the SHPO surface after boiling in a higher nanofluid concentration exhibited a contact angle of 20°, while after boiling in 0.001 wt.%, an average apparent contact angle of 79° was measured. Hydrophilic porous boiling interface induces an increase in liquid supply towards dry spots during bubble departures, universally enhancing the critical heat flux by mitigating dryout spots [57,58]. Nonetheless, in comparison with their superhydrophilic counterparts, superhydrophobic surfaces manifested lower CHF values after five consecutive runs at both tested concentrations. This observation aligns with findings from boiling studies on superhydrophilic and superhydrophobic surfaces with water, where it is well-established that superhydrophilic surfaces tend to delay dryout and augment CHF, whereas poorly wettable surfaces (superhydrophobic) typically fail to impede bubble coalescence, leading to the transition into film boiling at lower heat flux values [43,59,60].

**Figure 11:**
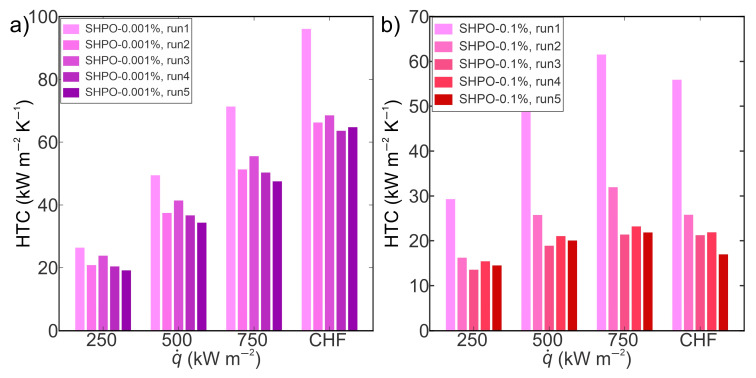
Heat transfer coefficients of TiO_2_–water nanofluid on a superhydrophobic laser-textured surface at (**a**) 0.001 wt.% and (**b**) 0.1 wt.% concentrations of TiO_2_ nanoparticles.

The HTC values during nanofluid boiling on superhydrophobic surfaces at various heat flux levels are presented in Figure 11. The highest HTCs were observed during the first run at a heat flux near the CHF for both tested concentrations. Specifically, at 0.001 wt.%, the maximum HTC reached 96 kW m^−2^ K^−1^, signifying an 88% enhancement over its superhydrophilic counterpart. Subsequent experimental runs revealed a decrease in HTC values towards the fifth run at both concentrations, attributed to nanoparticle deposition on surfaces. The deposition of nanoparticles in laser-induced microcavities resulted in decreased nucleation site density, thereby deteriorating the heat transfer coefficient. At 0.1 wt.%, the most substantial decrease in HTC, 75% from the first to the fifth run, was observed, which is 10% less than that observed on the superhydrophilic counterpart. The decrease in HTC on superhydrophobic surfaces can be ascribed to the hydrophobic coating, potentially mitigating nanoparticle deposition on the laser structure, thereby lowering the decrease in nucleation site density and lowering the HTC deterioration. A detailed discussion on the impact of superhydrophobic coating on the mitigation of nanoparticle deposition onto structured surfaces is provided in the subsequent section. Furthermore, a comparative analysis of results obtained with nanofluids on superhydrophobic surfaces and water on reference surfaces reveals improvements in HTC and CHF of up to 90% and 20%, respectively. This enhancement is predominantly attributed to laser-induced cavities on surfaces rather than the addition of nanoparticles to the base fluid.

**Figure 12:**
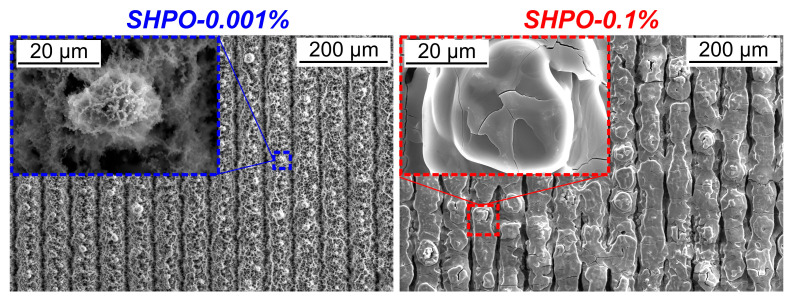
SEM images of superhydrophobic samples after boiling at concentrations of 0.001 wt.% (**left**) and 0.1 wt.% (**right**).

Furthermore, SEM images presented in Figure 12 show that the deposition of a thicker layer of nanoparticles occurred on the superhydrophobic (SHPO) sample subjected to boiling at a higher concentration, in contrast to the sample subjected to a lower concentration. The observed augmentation in the thickness of the TiO_2_-deposited nanoparticle layer on superhydrophobic surfaces is concomitant with an increase in nanoparticle concentration, which is an analogous trend observed in superhydrophilic samples. As previously mentioned, the larger thickness of the nanoparticle layer may induce greater thermal resistance, thereby resulting in a more pronounced degradation of boiling performance.

### 3.4. Nanoparticle Deposition and Its Influence on Boiling Heat Transfer

**Figure 13:**
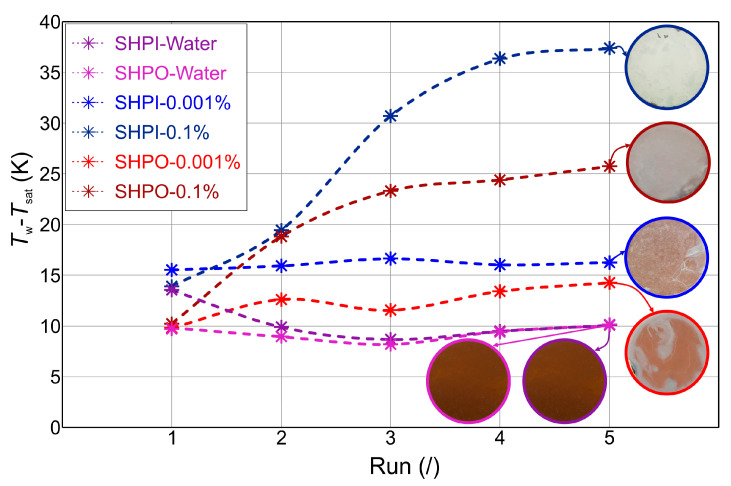
Nanoparticle deposition-induced changes, characterized by mean pool boiling curve wall temperature in the heat flux range of 50–900 kW m^−2^ and corresponding sample surface images. Plots of samples boiled in clean water are included to serve as a reference and point out that boiling performance also changes in clean water, where no deposition was observed.

From observations noted in previous sections, one can observe the similarities between the boiling of nanofluids and particulate fouling during nucleate pool boiling. Clearly, the local deposition of nano-sized particular matter onto the modified topography plays a key role in measurable changes in overall pool boiling performance with time. The latter can best be characterized by a shift of the pool boiling curve to higher superheats, which is to an extent also influenced by: (i) the stochastic nature of nucleation site activation/deactivation after each run; (ii) the CHF-induced changes to surface morphology (note the improvement in performance of the SHPI sample in water after the first CHF incipience due to oxide transformations); and (iii) the dynamic testing procedure affecting deposition. Hence, to reduce these influences, the mean wall superheat was computed in the heat flux region between 50 kW m^−2^ and 900 kW m^−2^ for all tested surface-fluid combinations (see Figure 13). In this way, one can more accurately compare the boiling curve shifts, which are indicative of performance degradation (or improvement) and thus correlated to nanoparticle deposition. The values clearly show the impact of nanoparticle deposition (or lack thereof) on surface temperature changes for all the tested surface-fluid combinations. The SHPI and SHPO samples exhibited a small shift of −3.5 K and +0.2 K after the five tests in pure water, respectively, which indicates that changes are not induced only by deposition. However, in the absence of a deposit-forming element, these shifts are minimal or even beneficial (towards lower superheats). When nanoparticles are present, the smaller concentration of 0.001wt.% appears to influence the SHPO surface to a larger extent (+4.5 K shift, compared to the +1.2 K shift of the SHPI sample), which may originate from the more pronounced change of wetting of the SHPO surface. The transition towards its improved wetting points to an increase in surface energy, which increases the energy barrier for nucleation and results in bubble formation at a higher surface superheat [61]. On the other hand, the SHPI sample’s wettability changes to a much smaller extent. The latter observations indicate that, at very low nanoparticle concentrations, the dominant influence on boiling curve shifts appears not to be the added thermal resistance of the deposit but instead the change in wetting properties, correlated to the deposit accumulation and oxide growth.

On the contrary, when both samples were tested in a nanofluid of 0.1 wt.%, the respective shifts of +23.5 K and +15.5 K were much more pronounced, pointing towards a larger and dominant effect of added thermal resistance, originating from a thick deposit layer that covered the surface (see SEM micrographs in Figure 9 and Figure 12). In respect to this, the SHPO surface degraded slower and to a smaller extent, pointing towards improved mitigation of surface deposition, as also evident by less surface discoloration (see sample images in Figure 13). This may be attributed to the weaker adhesion of particles to the SHPO sample with low surface energy, as well as to the distinct features of superhydrophobic nano- and microstructured surfaces. Namely, the trapped gas and vapor content within such surface structures is much higher than within their unhydrophobized counterpart, acting as a mitigative blanket against deposition. The increased affinity towards the gaseous phase promotes bubble nucleation, resulting in vigorous lateral and vertical coalescence of many small and fast-forming bubbles over the surface. Consequently, near-wall turbulence and shear stress are increased, which has previously been shown to mitigate particulate deposition [19].

The discussion above is especially relevant when considering a combination of nanoparticles and boiling surface materials, where the latter has a higher thermal conductivity than the former, such as the combination used in this study. In such cases, it is critical to mitigate the deposition of nanoparticles as much as possible to avoid inducing a large thermal resistance. In contrast, if highly conductive nanoparticles were used, the deposition-mitigative feature would likely have a smaller influence, which will be the focus of our future studies.

## 4. Conclusions

The pool boiling performance of TiO_2_–water nanofluids at two distinct concentrations (0.001 wt.% and 0.1 wt.%) was studied on laser-engineered surfaces in superhydrophilic and superhydrophobic states. The following conclusions were drawn based on the obtained experimental results:

1. Superhydrophobic and superhydrophilic laser-engineered surfaces exhibited an HTC enhancement of up to 120% over the reference surface due to fabricated microcavities that led to increased nucleation site density.
2. The boiling of nanofluids on functionalized (i.e., superhydrophilic and superhydrophobic laser-engineered) surfaces failed to provide an additional improvement in the HTC compared to boiling pure water.
3. At a low nanoparticle concentration, the influence of nanofluid on boiling performance is minimal, with CHF values comparable to those obtained using pure water on both the untreated, superhydrophilic and superhydrophobic laser-textured surfaces. On the other hand, at a high nanoparticle concentration, superhydrophobic and superhydrophilic laser-textured surfaces exhibited CHF enhancement up to 30% over their counterparts tested with pure water.
4. Boiling performance changes can be connected to nanoparticle deposition on the surface, where the superhydrophobic surface outperforms its non-hydrophobized counterpart at high concentrations. The influence of deposit-induced added thermal resistance is smaller at low nanofluid concentrations, and instead, the changes appear to be governed by a deposit-induced wettability transition.

## Figures and Tables

**Table 1:** Summary of tested working fluids and surfaces within this study.

Sample Name	Working Fluid	Concentration (wt.%)	Static Contact Angle (°)	
			Before Boiling	After Boiling
REF	Water	/	61	93
SHPI	Water	/	0	110
SHPO	Water	/	164	148
SHPI-0.001%	TiO_2_–water	0.001	0	17
SHPI-0.1%	TiO_2_–water	0.1	0	0
SHPO-0.001%	TiO_2_–water	0.001	164	79
SHPO-0.1%	TiO_2_–water	0.1	164	20

**Table 2:** Measurement uncertainty.

Heat Flux Level (kW m^−2^)	Heat Flux (%)	Heat Transfer Coefficient (%)	Surface Superheat (K)
250	9–11	10–13	0.4
1000	5	12–16	0.8

## Data Availability

Data are available from the authors upon reasonable request.
